# Advanced Ovarian Dysgerminoma With Supraclavicular Lymph Node Metastasis and Peritoneal Carcinomatosis: A Rare Case Report

**DOI:** 10.1002/ccr3.72503

**Published:** 2026-04-12

**Authors:** Bishal Khaniya, Sushant Shah, Dhiraj Adhikari, Abhaya Acharya, Aashish Poudel, Suresh Gautam

**Affiliations:** ^1^ Tribhuvan University Institute of Medicine Maharajgunj Medical Campus Kathmandu Nepal; ^2^ Chitwan Medical College Bharatpur Chitwan Nepal

**Keywords:** BEP chemotherapy, FIGO stage IVB, germ cell tumor, ovarian dysgerminoma, peritoneal carcinomatosis, supraclavicular lymph node metastasis

## Abstract

Ovarian dysgerminoma is a rare, highly chemo‐sensitive germ cell tumor usually presenting early. We report a FIGO stage IVB case with supraclavicular lymph node metastasis and peritoneal carcinomatosis in a 31‐year‐old woman. Diagnosis was based on classical histopathology. Cytoreductive surgery followed by BEP chemotherapy achieved a favorable clinical outcome.

## Key Clinical Message

Advanced ovarian dysgerminoma may rarely metastasize to supraclavicular lymph nodes and peritoneum. Markedly elevated LDH with normal AFP can aid early suspicion. Histopathology confirms diagnosis when IHC is unavailable, and cytoreductive surgery followed by BEP chemotherapy can achieve durable remission, even in FIGO stage IVB disease.

## 1 Introduction

Ovarian dysgerminoma is a rare malignant germ cell tumor arising from ovarian primordial germ cells. It represents approximately 32%–33% of all malignant ovarian germ cell tumors and fewer than 5% of all ovarian malignancies [1]. Unlike epithelial ovarian cancers, dysgerminomas lack well‐defined precursor lesions and have an unclear pathogenesis [2]. The tumor predominantly affects adolescents and young adults, peaking between 16 and 20 years, and is uncommon in postmenopausal women [2]. Although usually unilateral, bilateral involvement occurs in approximately 10%–15% of cases, typically in advanced disease (FIGO Stage III–IV) [3].

Clinically, dysgerminomas often present as large, rapidly enlarging abdominal masses with nonspecific symptoms such as pain, distension, weight loss, and fever [4]. Radiologically, they appear as solid, lobulated pelvic masses with enhancing fibrovascular septa on contrast imaging [5]. Histologically, they consist of large polygonal tumor cells with clear cytoplasm and prominent nucleoli, separated by fibrous septa infiltrated by lymphocytes [6].

Tumor markers are valuable both diagnostically and for surveillance. LDH is typically elevated due to high tumor metabolic activity, while β‐hCG and CA‐125 may be mildly raised in some cases [7]. Management depends on the disease stage and fertility considerations. Fertility‐sparing surgery is appropriate for Stage IA tumors, whereas advanced stages require radical cytoreductive surgery followed by adjuvant BEP chemotherapy [4].

We present a rare case of FIGO Stage IVB bilateral ovarian dysgerminoma in a 31‐year‐old woman with supraclavicular lymph node metastasis and peritoneal carcinomatosis—a presentation of notable rarity given the tumor's typically localized presentation and predictable lymphatic spread.

This case report has been prepared in accordance with the SCARE 2025 guidelines [8].

## 2 Case Presentation

### 2.1 Patient Information

A 31‐year‐old woman (gravida 1, para 1, abortion 1) presented with a three‐month history of progressive abdominal distension, diffuse abdominal pain, and intermittent low‐grade fever. She had no significant past medical or surgical history and no known family history of malignancy. There was no history of weight loss, bowel or urinary symptoms, or prior gynecological illness.

### 2.2 Clinical Findings

On presentation, the patient was conscious, oriented, and hemodynamically stable, with a blood pressure of 100/60 mmHg, heart rate of 80 beats per minute, and body temperature of 36.7°C. General physical examination revealed pallor but no peripheral edema or palpable superficial lymphadenopathy. Abdominal examination showed marked distension with prominent superficial veins. A fixed, firm, deep‐seated central abdominal mass measuring approximately 20 × 10 cm was palpable. Shifting dullness was present, suggestive of ascites. Pelvic examination could not clearly delineate the adnexal structures due to the large mass.

### 2.3 Diagnostic Assessment

#### 2.3.1 Laboratory Investigations

Routine hematological investigations were within normal limits except for thrombocytosis. Serum uric acid levels were reduced. Tumor marker evaluation revealed elevated β‐human chorionic gonadotropin (β‐hCG) levels of 476.45 IU/L, markedly elevated CA‐125 levels of 818.5 U/mL, and significantly raised lactate dehydrogenase (LDH) levels exceeding 1995 IU/ *L. alpha* ‐fetoprotein (AFP) levels were within normal limits (4.29 ng/mL).

#### 2.3.2 Imaging Studies

**FIGURE 1:**
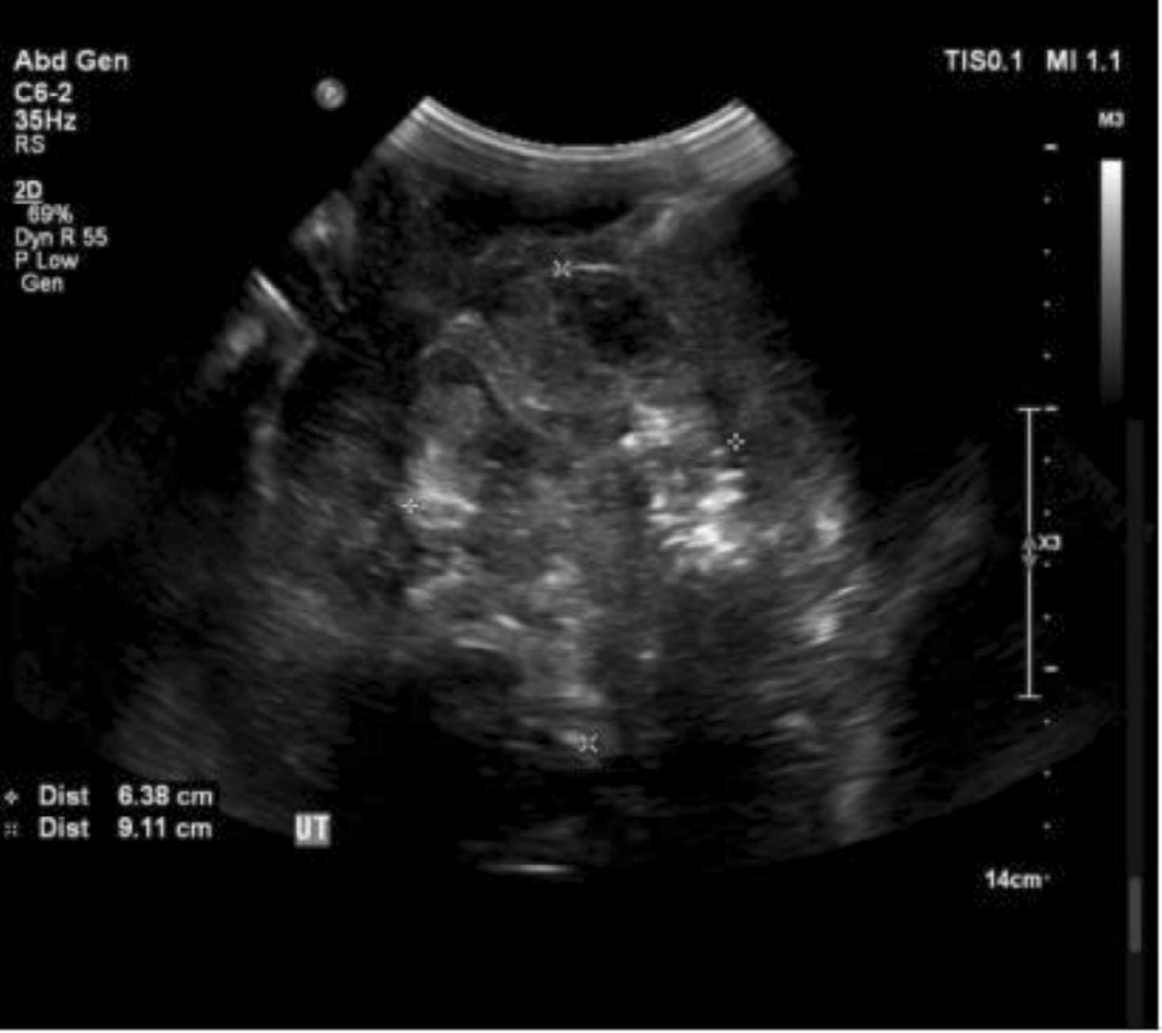
Ultrasonography showing a large echogenic abdominopelvic mass crossing the midline.

**FIGURE 2:**
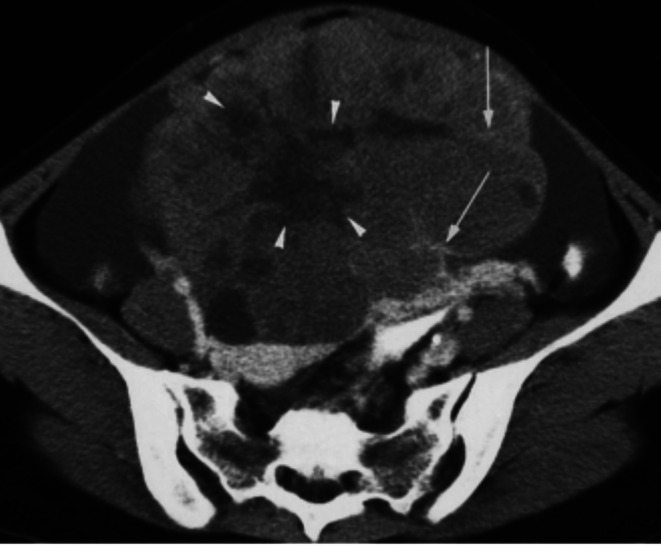
Contrast‐enhanced CT showing a large multilobulated solid ovarian mass with enhancing fibrovascular septa (arrows) and cystic areas (arrowheads).

**FIGURE 3:**
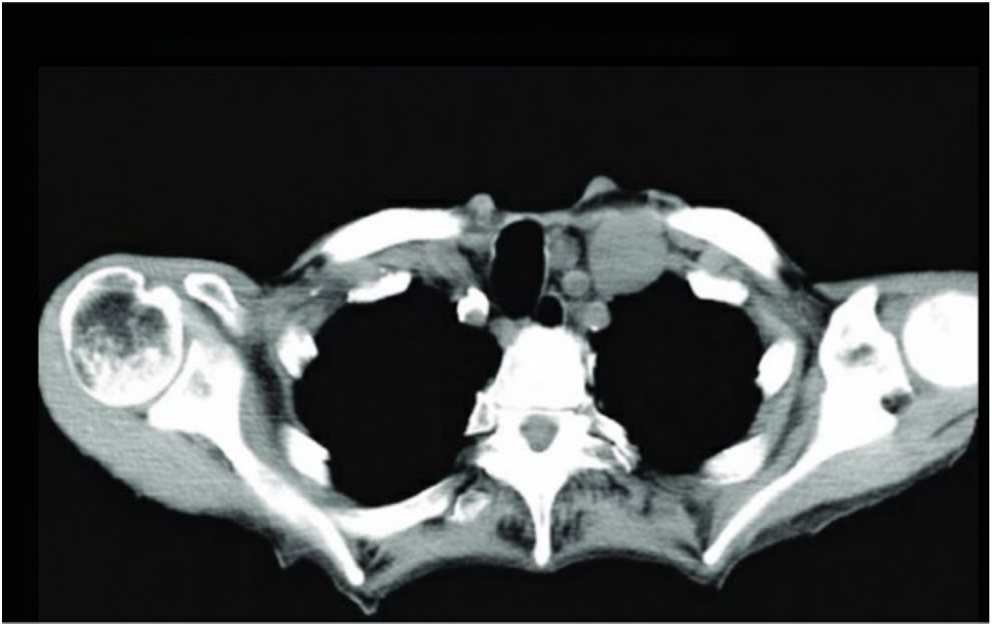
Left supraclavicular lymph node demonstrating metastatic malignant germ cell tumor.

**FIGURE 4:**
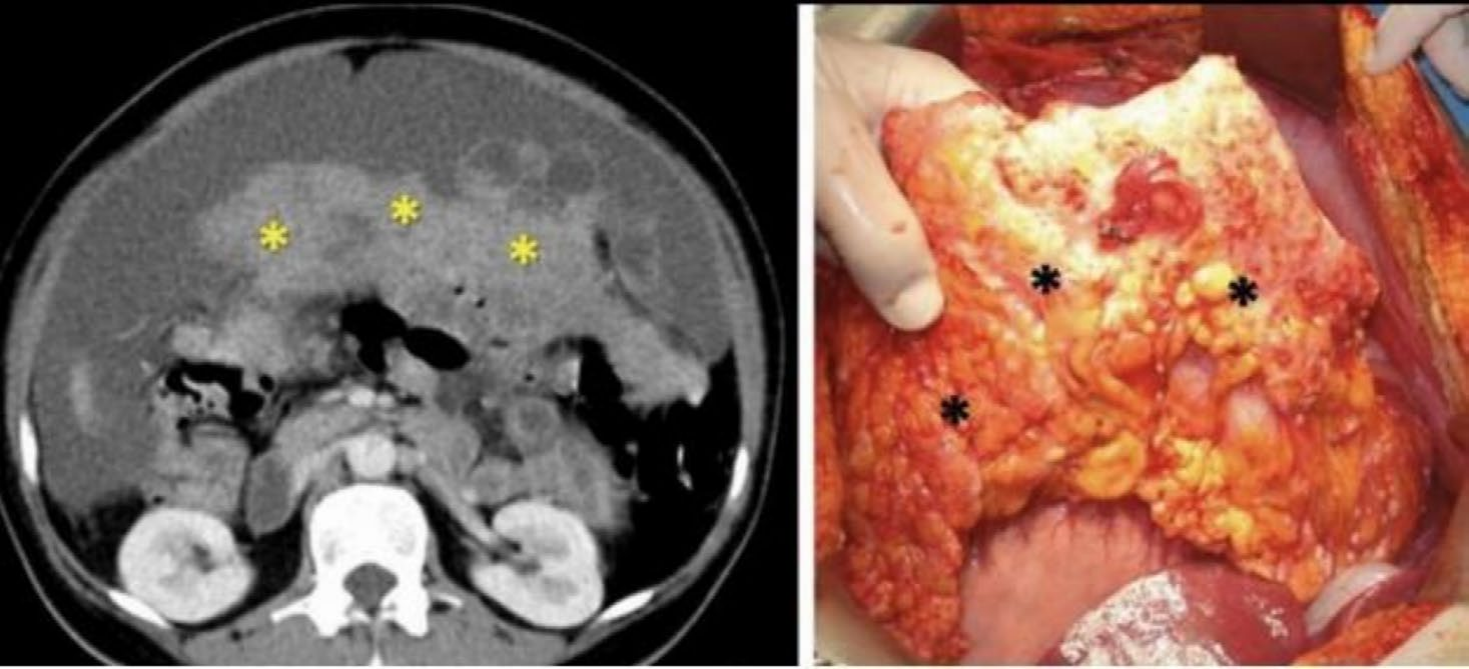
Contrast‐enhanced CT showing peritoneal carcinomatosis with ascites (left) along with the corresponding gross intraoperative appearance.(right).

Abdominal and pelvic ultrasonography revealed a large echogenic abdominopelvic mass crossing the midline, associated with moderate ascites (Figure 1). Contrast‐enhanced computed tomography (CT) of the abdomen and pelvis demonstrated a large multilobulated solid ovarian mass measuring approximately 25 × 20 cm, involving both ovaries, with enhancing fibrovascular septa and areas of cystic degeneration (Figure 2). CT imaging also showed extensive peritoneal deposits with omental caking, liver surface metastases, and gross ascites consistent with peritoneal carcinomatosis (Figure 4). Contrast‐enhanced CT of the neck revealed an enlarged left supraclavicular lymph node suspicious for metastatic involvement (Figure 3), suggesting distant lymphatic dissemination.

#### 2.3.3 Cytological Evaluation

Fine‐needle aspiration cytology (FNAC) from an enlarged left supraclavicular lymph node revealed malignant cells consistent with a metastatic germ cell tumor, indicating distant lymphatic dissemination.

### 2.4 Therapeutic Intervention

#### 2.4.1 Surgical Management

Following multidisciplinary tumor board discussion, the patient underwent exploratory laparotomy with comprehensive surgical staging and cytoreduction. The procedure included total abdominal hysterectomy, bilateral salpingo‐oophorectomy, infracolic omentectomy, pelvic and para‐aortic lymph node dissection, peritoneal biopsies, and tumor debulking. Given bilateral ovarian involvement, extensive peritoneal disease and distant nodal metastasis, fertility‐sparing surgery was not oncologically feasible.

#### 2.4.2 Intraoperative Findings

Intraoperatively, a large left ovarian mass measuring approximately 25 × 20 cm was identified, with stretching of the left fallopian tube (Figure 4). The right ovary appeared grossly normal but was removed for staging purposes. There was extensive peritoneal carcinomatosis with multiple tumor deposits over the peritoneal surfaces and liver capsule. Approximately 2 L of blood‐stained ascitic fluid was present. Multiple pelvic and para‐aortic lymph nodes were enlarged. Frozen section analysis confirmed metastatic tumor deposits.

#### 2.4.3 Histopathological Findings

**FIGURE 5:**
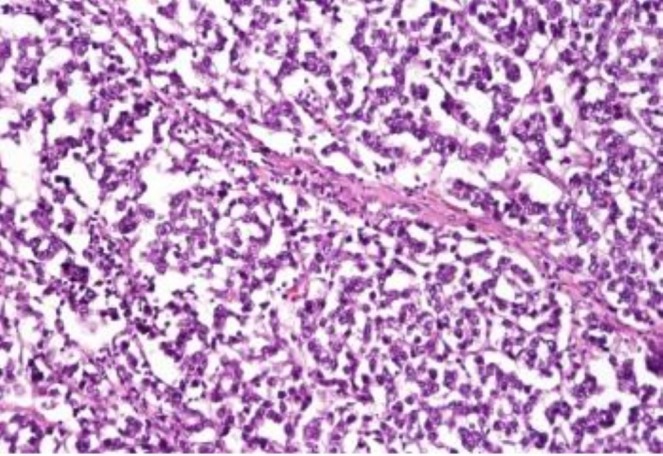
Histopathology (40×) showing sheets of large polygonal tumor cells with clear cytoplasm and prominent nucleoli separated by fibrous septa infiltrated by lymphocytes, consistent with dysgerminoma.

Histopathological examination revealed sheets of large polygonal tumor cells with clear cytoplasm and prominent nucleoli, separated by fibrous septa infiltrated by lymphocytes, consistent with dysgerminoma (Figure 5). Tumor involvement was confirmed in both ovaries, peritoneum, omentum, and resected lymph nodes. Although immunohistochemistry (OCT3/4, PLAP, CD117) is recommended for confirmation, the diagnosis in this case was based on classical histomorphological features.

### 2.5 Diagnosis

Based on clinical, radiological, cytological, and histopathological findings, a final diagnosis of FIGO Stage IVB bilateral ovarian dysgerminoma was established.

### 2.6 Postoperative Course and Follow‐Up

The postoperative period was uneventful. The patient received four cycles of adjuvant BEP chemotherapy (bleomycin, etoposide, and cisplatin). Follow‐up included monthly monitoring of serum LDH and β‐hCG levels during the first year, followed by bi‐monthly assessment in the second year. Surveillance imaging with CT or PET‐CT was planned every 3–6 months. Long‐term hormone replacement therapy was initiated following total abdominal hysterectomy and bilateral salpingo‐oophorectomy.

## 3 Discussion

Ovarian dysgerminoma, the most common malignant germ cell tumor of the ovary, typically occurs in young women with an excellent prognosis due to its chemosensitivity [4, 9]. While early‐stage disease demonstrates nearly 100% survival, even advanced stages achieve > 90% long‐term survival [9].

The progressive abdominal distension and pain over three months reflect the rapid growth characteristic of malignant ovarian germ cell tumors, which frequently attain large sizes before diagnosis. Associated fever and thrombocytosis likely represent a tumor‐driven inflammatory response secondary to extensive peritoneal involvement. Markedly elevated LDH and CA‐125 levels correlated with high tumor burden, ascites, and peritoneal irritation rather than tumor specificity. Imaging findings of large bilateral ovarian masses with ascites were consistent with advanced‐stage malignant ovarian germ cell disease.

To our knowledge, very few cases of ovarian dysgerminoma with supraclavicular lymph node metastasis have been reported, and the coexistence of distant nodal disease with peritoneal carcinomatosis is exceptionally rare. This case expands the known spectrum of metastatic behavior in ovarian dysgerminoma and challenges the conventional understanding of its dissemination patterns.

This case represents FIGO Stage IVB disease due to supraclavicular lymph node metastasis and peritoneal carcinomatosis—sites rarely involved in dysgerminoma [10]. Metastasis usually follows lymphatic spread to pelvic and para‐aortic nodes [7]; distant spread beyond the diaphragm is exceptional [11]. Such atypical dissemination may relate to aggressive tumor biology or KIT mutations, reported in a subset of dysgerminomas [10].

Serum LDH (> 1995 IU/L) was markedly elevated, correlating with tumor burden and serving as a reliable marker for diagnosis and therapeutic response [7]. Mild elevation of β‐hCG supported a germ cell origin, while CA‐125 elevation was likely secondary to extensive peritoneal irritation and ascites [4]. Although the therapeutic role of lymphadenectomy in malignant ovarian germ cell tumors remains debated [12], it remains crucial for accurate staging and prognostication. In this case, positive nodes confirmed the advanced stage and guided adjuvant therapy.

BEP chemotherapy remains the cornerstone of management for all stages ≥ IB [13]. Platinum‐based regimens have dramatically improved survival, achieving durable remission even with residual disease [14, 15]. Nevertheless, long‐term toxicities require vigilance: bleomycin‐related pulmonary fibrosis [16, 17], cisplatin‐induced nephro‐ and neuro‐toxicity [15], and etoposide‐related secondary leukemia [15]. Fertility preservation is a crucial consideration. Even after BEP chemotherapy, most women retain ovarian function [15]; however, in advanced bilateral disease, pre‐treatment fertility counseling and cryopreservation should be discussed [18, 19, 20].

Advanced‐stage dysgerminoma presenting with distant lymph node metastasis remains exceptionally rare and continues to challenge the conventional understanding of metastatic patterns in germ cell tumors [21, 22]. Recent molecular studies point out that alterations in pathways involving KIT and OCT3/4 might be associated with more aggressive tumor biology and increased metastatic potential in dysgerminomas [23]. The presence of peritoneal carcinomatosis, although unusual in a pure dysgerminoma, could indicate either a delay in clinical presentation or inherently rapid proliferation of the tumor that is capable of widespread serosal dissemination [24].

Supraclavicular lymph node involvement from ovarian tumors is presumed to occur via the thoracic duct and thus may provide a plausible explanation for this patient's atypical route of metastasis [25]. Advances in imaging suggest that PET‐CT has an increasingly important role in delineating occult metastases and assessing treatment response in malignant ovarian germ cell tumors, especially in advanced or ambiguous cases [26].

Tumor‐burden–reducing cytoreductive surgery plays a crucial role even in advanced disease, as reduction of disease volume improves chemosensitivity and treatment response [27]. Contemporary cohort studies confirm the superiority of BEP over alternative platinum‐based regimens for durable remission in metastatic germ cell tumors [28]. Long‐term follow‐up has also been very important since, while uncommon, late recurrences have been described well beyond five years, further emphasizing prolonged surveillance needs in dysgerminoma survivors [29].

Despite its educational value, this case report has several limitations. First, as a single‐patient report, it cannot establish causality or generalize findings to the broader population of patients with ovarian dysgerminoma. Second, molecular and genetic profiling, such as KIT or OCT3/4 mutation analysis, was not performed, which could have provided insights into the tumor's atypical metastatic behavior. Third, long‐term follow‐up data beyond the initial chemotherapy and early surveillance period are not yet available, limiting assessment of late recurrence, fertility outcomes, and long‐term treatment‐related toxicities. Finally, imaging modalities such as PET‐CT were not utilized preoperatively, which may have provided more precise staging and detection of occult metastases.

This case reinforces several important clinical lessons. Comprehensive staging, including evaluation of distant lymph nodes, is essential in all malignant ovarian germ cell tumors to ensure accurate assessment and optimal treatment planning. Multidisciplinary management plays a critical role in improving outcomes while minimizing morbidity. The inherent chemosensitivity of dysgerminoma means that the efficacy of chemotherapy often outweighs the impact of any residual disease, distinguishing it from epithelial ovarian cancers. Even in FIGO Stage IV disease, excellent long‐term prognosis is achievable with appropriate multimodal therapy. Finally, survivorship care must incorporate monitoring for long‐term treatment‐related toxicities and structured recurrence surveillance to ensure sustained remission and patient well‐being.

## 4 Conclusion

This report describes an exceptionally rare presentation of advanced ovarian dysgerminoma (FIGO Stage IVB) with supraclavicular lymphadenopathy and peritoneal carcinomatosis. Despite its aggressive metastatic profile, the tumor's inherent chemosensitivity enables favorable outcomes with appropriate multimodal management. This case emphasizes the importance of thorough metastatic evaluation, platinum‐based chemotherapy, and structured long‐term follow‐up in achieving durable remission and survival.

## Author Contributions

**Bishal Khaniya:** conceptualization, data curation, supervision, writing – review and editing. **Sushant Shah:** methodology, writing – original draft, writing – review and editing. **Dhiraj Adhikari:** supervision, writing – original draft, writing – review and editing. **Abhaya Acharya:** methodology, writing – original draft, writing – review and editing. **Aashish Poudel:** investigation, writing – review and editing. **Suresh Gautam:** writing – original draft, writing – review and editing.

## Funding

The authors have nothing to report.

## Ethics Statement

The case report did not intervene with the patient's treatment plan and hence it did not require ethical approval.

## Consent

Written informed consent was obtained from the patient for publication of this case report and accompanying images. A copy of the written consent is available for review by the editor‐in‐chief of this journal on request.

## Conflicts of Interest

The authors declare no conflicts of interest.

## Data Availability

The data that supports the findings of this study are available from the corresponding author upon reasonable request.
